# New erythrocyte parameters derived from the Coulter principle relate with red blood cell properties—A pilot study in diabetes mellitus

**DOI:** 10.1371/journal.pone.0293356

**Published:** 2023-10-26

**Authors:** Chloé Bourguignon, Clémentine Ansel, Jean-Philippe Gineys, Sophie Schuldiner, Damien Isèbe, Michael Geitner, Pierre Taraconat, Jean-Christophe Gris

**Affiliations:** 1 Department of Haematology, Nîmes University Hospital and University of Montpellier, Nîmes, France; 2 UMR UA11 INSERM - Montpellier University IDESP, Montpellier, France; 3 HORIBA Medical, Parc Euromédecine, Rue du Caducée, Montpellier, France; 4 Department of Metabolic Diseases and Endocrinology, Nîmes University Hospital, Nîmes, France; 5 Department of Gynaecology and Obstetrics, First Moscow State Medical University (Sechenov University), Moscow, Russian Federation; The Ohio State University, UNITED STATES

## Abstract

In routine hematological instruments, blood cells are counted and sized by monitoring the impedance signals induced during their passage through a Coulter orifice. However, only signals associated with centered paths in the aperture are considered for analysis, while the rejected measurements, caused by near-wall trajectories, can provide additional information on red blood cells (RBC), as recent publications suggest. To assess usefulness of two new parameters in describing alterations in RBC properties, we performed a pilot study to compare blood samples from patients with diabetes mellitus (DM), frequent pathological condition associated with impairment in RBC deformability, versus controls. A total of 345 blood samples were analyzed: 225 in the DM group and 120 in the control group. A diagram of R and P, the two new parameters derived from the analysis of impedancemetry pulses, was used to compare distribution of RBC subpopulations between groups. To discriminate RBC from DM and control individuals, based on our multiparametric analysis, we built a score from variables derived from R/P matrix which showed good performances: area under the receiving operating characteristic curve 0.948 (0.920–0.970), p<0.0001; best discriminating value: negative predictive value 94.7%, positive predictive value was 78.4%. These results seem promising to approach RBC alterations in routine laboratory practice. The related potential clinically relevant outcomes remain to be investigated.

## Introduction

In many hematology analyzers, blood cells are counted and sized by the Coulter principle [1]. The cells are enforced to flow in a micro-orifice, in which a strong electrical field is imposed by two electrodes, so that each time a cell crosses the aperture, an electrical pulse is measured. Counting pulses and assessing their maxima lead to the concentration and the volume distribution of the cells.

Measurement artefacts occur when cells flow along a trajectory in the vicinity of the aperture walls [2–4]. They are explained by the inhomogeneity of the electrical field and by complex cellular dynamics encountered in such regions. Measurement artefacts are known to skew the volume measurements, leading to a right-skewed size distribution of the cells [4]. This is why many efforts have been made over the last decades to remove pulses induced by near-trajectories. This may be done by implementing sample hydrodynamical focusing [4, 5]. A sheath flow enforces the cells to evolve in the core region of the detection area. A cost-effective alternative is the pulse-editing approach [4]: the spurious signals arising from near-wall paths are detected, then corrected or rejected before performing the analysis.

While signals induced by near-wall trajectories are unsuitable for sizing the cells, they may contain additional information on the latter. In a recent study, we showed that parameters derived from such signals are related to the shape and deformability of *in vitro* drug-treated red blood cells (RBC) [3]. Briefly, next to the micro-orifice walls, high velocity shears induce cells to rotate. This shear-induced rotation generates a peak on the electrical perturbation signal, whose waveform depends on distance to the wall. Two features calculated from the signal waveforms were introduced to characterize the cell trajectory in the measurement region: R (presence of a rotation peak) and P (time of the peak). Distribution patterns on R/P diagrams were found to vary when RBC were altered chemically with drugs known to spherize and to stiffen RBC. Since the cells alterations used for this proof of concept were artificial and likely far from what occurs physiologically, the following question naturally arises: are changes on R/P diagrams sensible enough for detecting pathological modifications of RBC occurring *in vivo*?

Indeed, alterations in RBC deformability (due to hemoglobinopathies or bio-mechanical membrane disorders) are found in several pathological states, which could be constitutive or acquired, transient or permanent [6–10]. This defective cell deformability could be involved in disease complications and severity prognosis [11–13]. However, most routine hematological analyzers used widely for complete blood count (CBC) do not allow approach of cell deformability. Such evaluation being historically limited to highly specialized techniques such as ektacytometry [14], even though recently developed devices are commercially available to measure RBC deformability [15, 16].

In order to test the potential clinical interest of R and P, we focused on one of the most prevalent diseases with an impaired globular deformability: diabetes mellitus (DM) [17]. Indeed, DM is a widespread metabolic dysfunction due to insulin resistance or deficiency, that leads to chronic hyperglycemia including chronic complications such as micro- and macro-angiopathy, with strong consequences on prognosis and health cost [18–22]. Impairment of red blood cell deformability is correlated with blood glucose concentration, glycated hemoglobin rate (HbA1c) but also creatinine plasma level, reflecting diabetic nephropathy [9, 13, 23, 24].

To test the hypothesis that alterations of RBC from patients with DM could be detected thanks to the original parameters derived from impedance pulses analysis (viz. R and P), we designed a pilot case-control study. A DM-associated positive conclusion would lead to study diagnostic and prognostic consequences, if any, on relevant clinical outcomes.

## Methods

### Study design and participants

To assess a potential association between RBC modifications observed in DM and these new physical parameters, a case control study was set up thanks to the collaboration between HORIBA Medical based in Montpellier (France) and the Department of Hematology of the University Hospital of Nîmes (France), with a specific support provided by the KIM MUSE IBS (Key Initiative Muse Interdisciplinary Blood Science), University of Montpellier.

During a 5 weeks-long recruitment period, all consecutive diabetic outpatients and inpatients with a routine blood count and glycated hemoglobin prescribed by physicians of the Department of Metabolic Diseases and Endocrinology (MME), University Hospital of Nîmes, were recruited in the patients’ group.

Serial healthy blood donors for transfusion at the Etablissement Français du Sang (EFS) were in parallel recruited in the controls’ group.

The study was performed in accordance with the policy on bioethics and human biological samples of French laws on clinical research and in accordance with the 1996 revised version of the 1975 Helsinki declaration. The study was approved by Nîmes University Hospital Institutional Review Board and ethics committee (IRB n◦ 22.01.21). According to their approval, a letter of information and of non-opposition to participate to this study was transmitted to patients. No written consent was required.

### Instruments

Analyses were performed on residual EDTA K3-anticoagulated blood samples initially collected for blood cell count as part of standard care. For both patients and controls, routine blood cell counts were performed on Sysmex XN^™^ hematology analyzer (Sysmex France, Villepinte, France). In addition, samples were analyzed with a mock-up derived from the ABX Micros 60 (HORIBA Medical), for the recording of the electrical pulses induced by RBC in an aperture-electrode system (also called Coulter-based system). The pulses acquisition is performed by a LabVIEW^™^ (National Instruments) code, as presented before [3, 4]. The mock-up differs from the commercial version ABX Micros 60, with an electronic bandwidth increased to 150 kHz. This modification guarantees a high-quality signal and a reliable electrical pulse processing. Commercial ABX Diluent was used (HORIBA Medical). Unlike some routine hematological analyzers, there is no need to spherize RBC prior acquisition and no hydrodynamical focusing is used with this device. All acquisitions were performed at room temperature. The original parameters derived from the pulse’s acquisitions are introduced in the following section.

### Definition of new parameters, derived from the pulse’s acquisitions

Pulse’s acquisitions computing and new parameters derived from these data have been extensively described elsewhere [2–4].

Briefly, a digital acquisition cleaning is performed prior to any pulse processing. This is done for removing most of unsuited signals that may be induced by cells coincidences in the aperture, white blood cells, platelets, or background noise.

**Fig 1:**
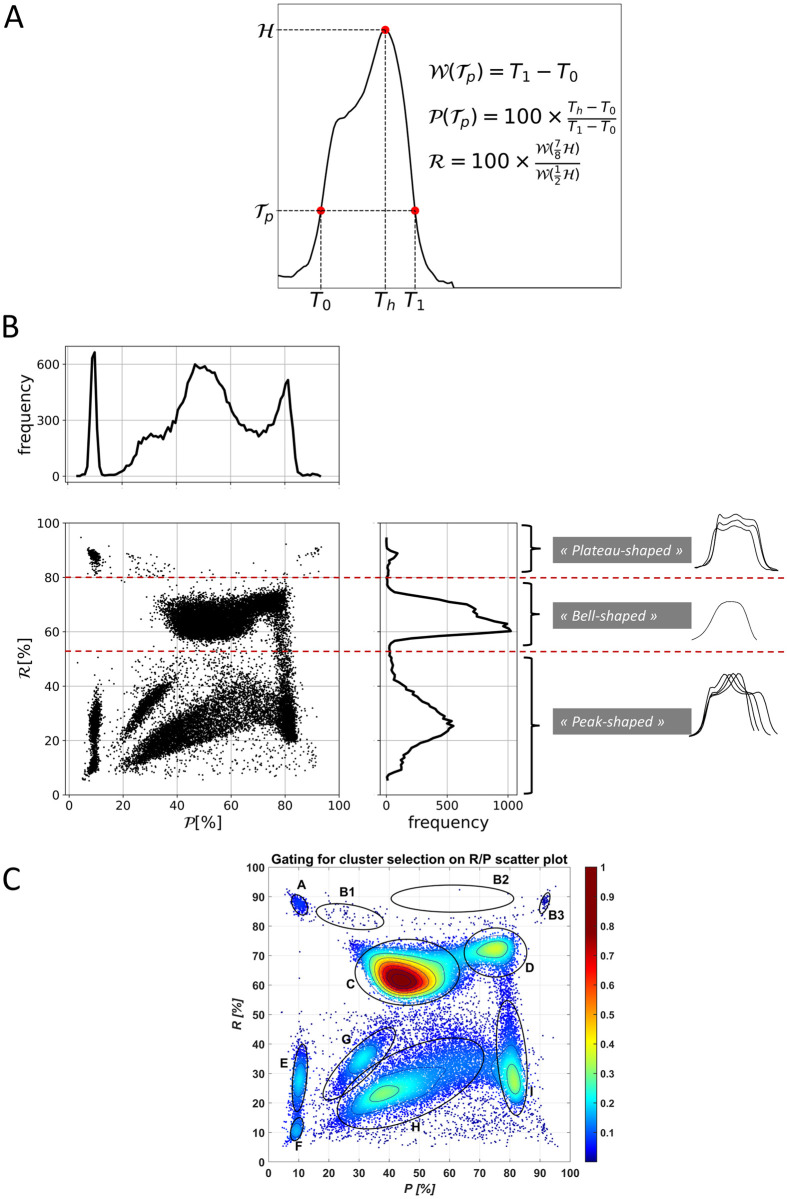
Definition of R and P, scatterplot and clustering derived from red blood cells pulse’s acquisitions. (a) Definition of quantities required for computing R and P. (b) *R/P* scatterplot and the three main subpopulations defined by thresholding on the metric R. (c) Clustering on R/P scatterplot.

The original parameters are based on quantities R and P. As a reminder, the metric R detects whether a rotation-associated peak is present on the pulse, while P assesses the moment at which the rotation, and thus the peak, occurs [3]. Let consider a curve representing an electrical pulse: H denotes the pulse maximum, while $W_{p}$ (T_1_-T_0_) refers to the pulse width (Fig 1a). The computation of P consists in assessing the relative position of the H projection along $W_{p}$. (T_H_) In this respect, P reads:

$$P=\;\frac{T_{h}-T_{0}}{T_{1}-T_{0}}\times 100$$

Note that P is parametrized by $T_{p}$, which corresponds to the acquisition threshold (equivalent to 13fL). As presented before [4], R writes:

$$R=\frac{W\left(\frac{7}{8}H\right)}{W\left(\frac{1}{2}H\right)}\times 100,$$

$W\left(\frac{7}{8}H\right)$ and $W\left(\frac{1}{2}H\right)$ are calculated at thresholds $\frac{1}{2}H$ to intersect the ascending and descending slopes of the pulses and $\frac{7}{8}H$ to intersect the rotation peak, if any.

A typical R/P diagram is shown in Fig 1b. Along the R axis, three interesting populations are visible:

- R≤50%: “Peak-shaped” pulses population, induced by cells that rotate due to velocity shear in the vicinity of the aperture edges,
- R>50% but ≤80%: “Bell-shaped” pulses population, originating from centered path of cells, aligned with the aperture axis [4],
- R>80%: “Plateau-shaped” pulses population, mainly represented by pulses with a small rotation-associated peak, *ie* without intersection between $\frac{7}{8}H$ and the peak. The later represents only a small percentage of the total acquisition, as the R distribution shows (Fig 1b).

Combining R with P provides a better overview of the trajectories. Indeed, the density plot makes appear more subpopulations (or clusters) than just “Peak-shaped”, “Bell-shaped” and “Plateau-shaped” (Fig 1c). In a nutshell, going from cluster C to cluster F covers the trajectories from the center to the edges of the aperture. Clusters A, B1, B2 and B3 belong to the “plateau shaped” population and are associated with near wall trajectories as well, but represent a small portion of the entire acquisition. Subpopulations observed in Fig 1c are defined with an automatic-gating method. First, an initial set (noted $G_{0}$) of 9 to 11 gates (according to the element quantities inside B1 and B2) is defined. On each gate $G_{0}$, a bidimensional binomial fit of the point distribution is performed as a function of R and P. For each point of the R and P matrix, a label is given according to the confidence ellipse calculated on each bidimensional probability function. If a point is in only one confidence ellipse, the related ellipse label is given to the point. If a point belongs to more than one confidence ellipse, the maximum probability is taken as label. Finally, if a point belongs to none of those confidence ellipses, the distance between the point and all the confidence ellipses is calculated and the closest ellipse label is given to the point. The eleven resulting populations are noted A, B1, B2, B3, C, D, E, F, G, H and I (Fig 1c).

### Statistical analysis

Quantitative data are presented as the median and interquartile range (IQR). Qualitative data are presented as values and percentages. Mann-Whitney test and *χ*^2^ tests were used for comparisons between group characteristics. Data of the matrix generated from R and P were evaluated first by univariate then by multivariate logistic regression analysis.

For multivariate models, a stepwise backward elimination was performed after selecting all variables identified by the univariate models as potential predictors at P < 0.15, with adjustment being finally performed for all variables with P < 0.15 in the multivariate models. In the case of two variables were strongly correlated (Pearson’s correlation coefficient > 0.80), a single variable was retained, based on feasibility criteria. The final model only included main effects with P < 0.10.

The estimated regression coefficients of the finally retained informative variables were used as the weighting coefficients of the parameters in the final simplified score describing the plausibility of being a diabetic patient rather than a control patient. The discriminating ability of this score was assessed by computing Receiver Operating Characteristic (ROC) curves and calculating the Area Under the Curve (AUC: C statistic). The best cut-off value was identified as the point maximizing the computed Youden index (sensitivity Se + specificity Sp—1). All tests were two-sided and assessed at the 5% significance level.

In absence of any available data allowing to calculate a number of subjects necessary to reach significance in that comparative pilot evaluation, the study was based on the number of individuals we were able to recruit during the study period.

Statistical analyses were performed using StatView^®^-windows software version 5.0 (SAS Institute Inc., Cary, NC, USA) and XLSTAT^®^ software version 2015.4.01.20116 (Addinsoft SARL, Paris, France).

## Results

### Participants characteristics

Between 6^th^ April and 15^th^ May 2021, 225 patients were included in the DM patient group. A total of 120 controls were recruited over the two following periods: between 25^th^ September and 12^th^ October 2020; between 8^th^ June and 15^th^ June 2021. Main characteristics results are presented in Table 1. According to the free and anonymous nature of blood donation in France, no demographic nor clinical information was available for controls but only age range (18–70 years old). Sex ratio of the DM patients was balanced. Median of blood count results was clinically comparable between groups, globally fitting in our normal laboratory ranges.

**Table 1:** Participant demographics and blood count characteristics.

	DM PATIENTS GROUP	CONTROL GROUP	p
**N**	225	120	
**Age (y/o)** Median (range)	61 (10–100)	N/A	
**Female sex, n (%)**	111 (49.3)	N/A	
**RBC (10^12^/L)**	4.5 (4.1–4.9)	4.5 (4.3–4.9)	0.2796
**Hemoglobin (g/L)**	132 (119–145)	139 (131–147)	< 0.0001
**MCV (fL)**	90 (86.8–93.2)	92.9 (90.1–95.6)	< 0.0001
**MCHC (g/L)**	32.6 (31.8–33.4)	33 (32.4–33.6)	0.0009
**RDW (%)**	9.93 (8.96–10.89)	(9.23–10.51)	0.0206
**Reticulocytes (10^9^/L)**	101.7 (65.3–138)	63.8 (51.7–75.9)	< 0.0001
**Platelets (10^9^/L)**	251 (203–299)	247 (213–281)	0.1969
**WBC (10^9^/L)**	7.58 (5.72–9.44)	6.09 (5.22–6.97)	< 0.0001

Blood count values are presented as mean (IQR). Abbreviations: RBC, red blood cell count; MCV, mean corpuscular volume; MCHC, mean corpuscular hemoglobin concentration; RDW, red cell distribution width; WBC, white blood cell count; y/o, years old; N/A, not available

### Signal analysis

The number of pulses acquired for analysis of each sample after the acquisition cleaning were very similar: 29,958 (28,880–31,036) for controls and 28,889 (26,351–31,428) for DM patients.

Overall, the P and R median values were significantly different between the two groups. P values were higher in the control group than in the DM group: 54.542% (53.741–55.343) versus 53.720% (52.998–54.442) (p<0.0001). R values were lower in controls than in DM patients: 58.502% (58.185–58.820) versus 58.579% (58.296–58.863), p = 0.003.

From the R/P matrix, univariate analysis results of signal populations distribution are presented in Table 2. All variables, except B1 and F, were positively (“Bell-shaped” pulses, “Plateau-shaped” pulses, A, B1, C and G percentages) or negatively (”Peak-shaped” pulses, B3, D, E, H and I percentages) associated with the DM status. B2 population was not statistically interpretable.

**Table 2:** Results of univariate and multivariate logistic regression analysis of R/P matrix sub-populations in their capacity to predict the diabetic status.

	VARIABLES	X^2^	OR (95% CI)	p
**UNIVARIATE**	RBC (10^12^/L)	2.096	0.742 (0.495–1.111)	0.1477
	Hemoglobin (g/L)	17.869	0.735 (0.637–0.848)	<0.0001
	MCV (fL)	17.534	0.901 (0.857–0.946)	<0.0001
	MCHC (g/L)	14.019	0.657 (0.528–0.819)	0.0002
	RDW (%)	15.815	1.675 (1.299–2.161)	<0.0001
	Reticulocytes (10^9^/L)	53.996	1.048 (1.035–1.061)	<0.0001
	Platelets (10^9^/L)	3.488	1.003 (1.000–1.006)	0.0618
	WBC (10^9^/L)	31.837	1.438 (1.267–1.631)	<0.0001
	"Bell-shaped" pulses (%)	72.224	13.61 (7.453–24.853)	<0.0001
	“Peak-shaped” pulses (%)	58.305	0.225 (0.153–0.329)	<0.0001
	"Plateau-shaped" pulses (%)	7.627	1.881 (1.201–2.946)	0.0058
	A (%)	9.528	2.48 (1.407–4.615)	0.002
	B1 (%)	1.644	5.231 (0.417–65.638)	0.1998
	B2 (%)	-	-	-
	B3 (%)	19.027	7.02 E^-6^ (3.39 E^-8^–0.001)	<0.0001
	C (%)	69.884	20.4 (10.059–41.371)	<0.0001
	D (%)	56.337	0.073 (0.037–0.144)	<0.0001
	E (%)	9.651	0.287 (0.131–0.631)	0.0019
	F (%)	0.087	0.925 (0.551–1.553)	0.768
	G (%)	8.387	2.842 (1.401–5.763)	0.0038
	H (%)	32.853	0.424 (0.316–0.569)	<0.0001
	I (%)	61.158	0.035 (0.015–0.081)	<0.0001
**MULTIVARIATE**	"Bell-shaped" pulses (%)	27.809	0.002 (2.46 E^-4^–0.022)	<0.0001
	“Peak-shaped” pulses (%)	26.503	0.051 (0.017–0.159)	<0.0001
	C (%)	51.793	470.863 (88.81–2517.13)	<0.0001
	I (%)	3.15	4.809 (0.849–27.25)	0.0759

Multivariate analysis: final adjustment is performed on all variables identified by the univariate models as potential predictors at P<0.15, the final model only including main effects with P<0.10. Abbreviations: RBC, red blood cell count; MCV, mean corpuscular volume; MCHC, mean corpuscular hemoglobin concentration; RDW, red cell distribution width; WBC, white blood cell count.

Some classical variables from the CBC were also associated with the DM status (hemoglobin, mean corpuscular volume (MCV), mean corpuscular hemoglobin concentration (MCHC), red cell distribution width (RDW), reticulocytes and white blood cell count (WBC)).

Finally, multivariate analysis led to retain four parameters being associated with the DM status: percentages of “Plateau-shaped” and”Peak-shaped” pulses, percentages of events in the C and I boxes (Table 2). None of the CBC variables remained significant.

### Score performances

**Fig 2:**
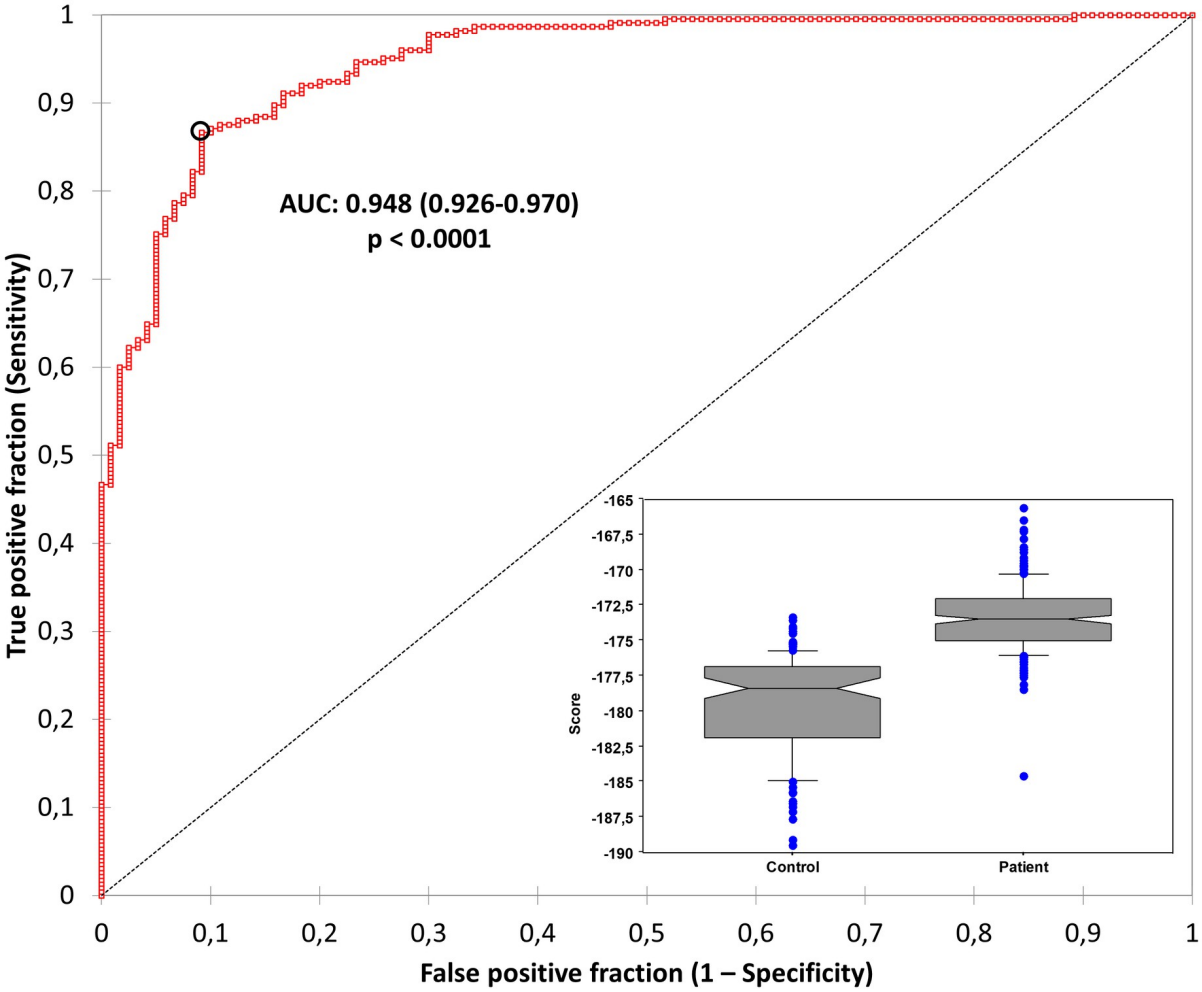
New parameters R and P to discriminate red blood cells from DM patients versus control group. Receiver operating characteristic curve analyzing discrimination power of the score derived from R/P matrix between DM patient group and control group. Abbreviations: AUC, area under the curve; ***, p<0.0001.

The score describing the plausibility of being a diabetic blood sample derived from the multivariate analysis was S = -(6.068 * % of “Bell-shaped” pulses) + (6.155 * % of pulses in box C) + (1.57 * % of pulses in box I)–(2.968 * % of “Peak-shaped” pulses). The value of its area under the computed receiving operating curve was 0.948 (0.926–0.970), p<0.0001 (Fig 2). The 0.881 S value had the following characteristics in predicting the diabetic status: sensitivity 0.867 (0.815–0.905), specificity 0.908 (0.841–0.949), negative predictive value 0.947 (negative likelihood ratio LR-: 0.147) and positive predictive value 0.784 (LR+: 9.455).

## Discussion

In this case control pilot study, we evaluated two promising new parameters, P and R. derived from the analysis of electronic signals generated by erythrocytes passing through the aperture of the Coulter counter. These parameters are designed for being implemented into classical erythrocytes parameters displayed by widespread CBC automates using Coulter principle.

Significant differences are observed for both P and R between DM and control samples. From the analysis of R/P matrix subpopulation distribution, we determined a multiparametric score showing good discriminative performances: 94.7% negative predictive value and 78.4% positive predictive value.

Parameters P and R characterize the rotation dynamics of the cell and are closely related to its trajectory in the aperture. Varying RBC properties in physiological ranges have shown negligible effects on the trajectory but found to modify the pulse shape [3]. We though that these changes result in variations in P and R, since they derive from the signal. Developing metrics specifically designed for capturing the pulse changes induced by impaired deformability would improve the discriminative performance. However, developing such parameter is not straightforward because of the huge variety of pulse signatures. Further work are required to gain a better understanding of the link between cell properties, trajectories, and pulse signatures.

As reported before from *in vitro* data [3], the deformability of RBC might participate to some extent to the differences observed between the two groups but still to be characterized. Indeed, one can’t confirm that RBC deformability is the dominating determinant of such changes until a proper correlation study is performed to strictly compare these parameters to ektacytometry, considered as the gold standard method to assess RBC deformability. Nonetheless, thanks to its derivation from the widespread used Coulter system and its apparently encouraging discriminative performances, analysis derived from R/P matrix seem promising to approach intrinsic RBC alterations in routine laboratory practice in DM patients. Further works are required to determine whether these new P and R parameters may represent clinically relevant outcomes in patients. In a more general way, their application in other diseases than DM in which RBC intrinsic properties impairment may participate to the clinical prognosis also has to be documented.

Among pathological disorders associated with acquired modifications of the RBC biomechanical properties, DM was selected due to the large prevalence of this disease, allowing facilitated enrollment for this first study applied to a human disease. Moreover, alteration in RBC deformability is broadly studied in DM, both in preclinical and clinical studies. When submitted to high glucose concentrations, RBC undergo various composition changes (for instance glycation of membrane proteins [25] and hemoglobin [26, 27]) that were found to impact their bio-mechanical properties [9, 13, 23, 28]. Indeed, *in vitro* studies [29] state a decreased RBC deformability, when they are suspended in glucose solutions. This reduction of the cells’ deformability appears to depend on both glucose concentration and incubation time. In addition, clinical studies employing different methods for assessing cells deformations have concluded on the reduced deformability of RBC in diabetes [13, 17, 23, 24, 28].

The operating regime of the instrument used for the pulse acquisitions is representative of many commercial automates. Hence, this approach would be adaptable to other commercial systems, provided they implement the Coulter principle without hydrodynamical-focusing. Indeed, hydrodynamical-focusing enforces cells to flow at the center of the aperture in order to reduce volumetry errors [5]. But that would avoid any cellular rotation in the sensing region and make the processing of R and P irrelevant. Considering a Coulter system without hydrodynamical-focusing, one recommends nevertheless to adjust the gating of R/P matrix subpopulations to the instrument.

Our study has limitations. It is a non-blinded, retrospective, monocentric study. It is an initial pure association study with a disease state versus controls, not with disease state among all diseases. We lacked clinical and demographical data for the control group, due to the anonymous nature of blood donation in France, some of the missing data being potential confounders for the observed differences between patients and controls. Diabetic patients are by themselves heterogeneous, both in terms of diabetes type, treatments, complications, comorbidities and duration of diabetes. Here also, some of these data may act as potential confounders on the observed differences. This will have to be specifically studied.

Our study also has some strengths. It is the first one describing the application of our new erythrocyte-related experimental laboratory parameters to the characterization of human blood samples. We were able to include a large panel of DM patients regularly monitored at the Nîmes University Hospital, using *in situ* installation of the acquisition system to limit delay and transport between blood sampling and their analysis. The results are rather encouraging and push to go further in the systematic study of the informative capacity of these new parameters.
